# Features of connectivity of default mode network depending on polymorphism of serotonin transporter gene (5-HTTLPR)

**DOI:** 10.18699/vjgb-26-30

**Published:** 2026-04

**Authors:** A.V. Bocharov, A.N. Savostyanov, S.S. Tamozhnikov, A.E. Saprygin, D.A. Lebedkin, E.A. Merkulova, G.G. Knyazev

**Affiliations:** Scientific Research Institute of Neurosciences and Medicine, Novosibirsk, Russia; Scientific Research Institute of Neurosciences and Medicine, Novosibirsk, Russia; Scientific Research Institute of Neurosciences and Medicine, Novosibirsk, Russia; Scientific Research Institute of Neurosciences and Medicine, Novosibirsk, Russia; Scientific Research Institute of Neurosciences and Medicine, Novosibirsk, Russia; Scientific Research Institute of Neurosciences and Medicine, Novosibirsk, Russia; Scientific Research Institute of Neurosciences and Medicine, Novosibirsk, Russia

**Keywords:** EEG, 5-HTTLPR, serotonin transporter polymorphism, default mode network, resting-state networks, connectivity, ЭЭГ, 5-HTTLPR, полиморфизм гена транспортера серотонина, дефолтная сеть мозга, дефолт-система мозга, коннективность, сети покоя

## Abstract

Serotonin transporter gene polymorphism is important in the regulation of the serotoninergic system that affects mood and the regulation of emotions and behavior. In this study, 128 channel electroencephalogram recordings were performed, and buccal epithelium samples were obtained from 53 volunteers (32 females). La, Lg, and S alleles were identified by polymerase chain reaction. The aim of the study was to investigate the connectivity of the default mode network, measured using resting state electrophysiologic data, depending on the serotonin transporter gene polymorphism. Localization of the sources of bioelectrical activity of the cerebral cortex was performed by the beamformer method. Comparisons of LaLa genotype carriers and S or Lg allele carriers were performed using T-contrast of connectivity indices calculated between the nodes of the default mode network and the rest of the brain. It was found that carriers of the S allele were characterized by increased connectivity of the default mode network with the visual association cortex and with structures forming the posterior node of the default mode network, as well as increased connectivity of the posterior node of the default mode network with the right parahippocampal gyrus, and this pattern of connectivity may predispose to the onset and/or maintenance of intrusive thoughts. Whereas carriers of the LaLa genotype had higher connectivity of the anterior node of the default mode network with the right ventromedial prefrontal cortex, with the medial frontal gyrus, and with the posterior cingulate cortex, which is the structure of the posterior node of the default mode network, compared to carriers of the S or Lg allele. Also, carriers of the LaLa genotype had higher connectivity of the posterior node of the default mode network with the cluster involving the right dorsolateral prefrontal cortex compared to carriers of the S or Lg allele. It could be hypothesized that increased connectivity of the default mode network with brain structures (i. e., dorsolateral and ventromedial prefrontal cortex) involved in cognitive regulation processes may contribute to the regulation of the processes of the default mode network associated with autobiographical memory.

## Abstract (ru)

Полиморфизм гена транспортера серотонина имеет большое значение в регуляции серотонинергической системы, влияющей на настроение и регуляцию эмоций и поведения. В исследовании была проведена запись 128-канальной электроэнцефалограммы и взяты образцы буккального эпителия у 53 добровольцев (32 женщины). Аллели La, Lg и S были определены методом полимеразной цепной реакции. Целью работы было изучение особенностей коннективности дефолтной сети мозга, измеренной с помощью электрофизиологических данных состояния покоя, в зависимости от полиморфизма гена транспортера серотонина. Локализация источников биоэлектрической активности коры мозга осуществлялась методом формирователя пучка. Сравнение групп носителей LaLa генотипа и носителей одного из аллелей S или Lg было выполнено с помощью Т-контраста показателей коннективности, рассчитанных между узлами дефолтной сети мозга и остальным мозгом. Обнаружено, что для носителей S или Lg аллеля характерна повышенная коннективность дефолтной сети мозга со зрительной ассоциативной корой и со структурами, образующими задний узел дефолтной сети мозга, а также повышенная коннективность заднего узла дефолтной сети мозга с правой парагиппокампальной извилиной, что может предрасполагать к возникновению и/или поддержанию навязчивых мыслей. У носителей LaLa генотипа по сравнению с носителями S или Lg аллеля была выше коннективность переднего узла дефолтной сети мозга с правой вентромедиальной лобной корой, с медиальной лобной извилиной и с задней поясной корой, являющейся структурой заднего узла дефолтной сети мозга. Также у носителей LaLa генотипа по сравнению с носителями S или Lg аллеля была выше коннективность заднего узла дефолтной сети мозга с кластером, охватывающим правую дорсолатеральную префронтальную кору. Можно предположить, что увеличение коннективности дефолтной сети мозга со структурами мозга (дорсолатеральная и вентромедиальная префронтальная кора), участвующими в процессах когнитивной регуляции, способствует регуляции процессов дефолтной сети мозга, связанных с автобиографическими воспоминаниями

## Introduction

A large number of studies using functional magnetic resonance imaging (fMRI) have found a characteristic pattern of activation of brain structures forming a network at rest. This network was named the default mode network (DMN), and among the structures that comprise it are the medial prefrontal cortex, posterior cingulate cortex, precuneus, medial, lateral, and inferior parietal lobes (Raichle et al., 2001). It has been repeatedly shown that the DMN exhibits a sustained decrease in activity/connectivity during most externally oriented tasks requiring concentration on external stimuli. However, in the resting state, when attention is not focused on the external world, an increase in activity/connectivity has been observed in DMN structures (Raichle, 2015). Further studies have greatly expanded the understanding of DMN functions. For example, patterns of increased DMN activity/connectivity have been identified during self-referential information processing and retrieval of autobiographical memories, when reflecting on self and relationships with loved ones, when planning for the future, and when experiencing internal emotional states (Raichle et al., 2001; Knyazev, 2013; Raichle, 2015; Buckner, DiNicola, 2019; Hutchinson-Wong et al., 2024; Si et al., 2025). In addition, DMN dysfunction has been found to be associated with a number of psychiatric disorders (Hutchinson-Wong et al., 2024). Thus, in people with depression symptoms and in patients with major depressive disorder (MDD), there is an increase in DMN activity/connectivity, which may be associated with disturbances in the processes of self-reflection and emotional regulation (Berman et al., 2011; Hamilton et al., 2011, 2013; Knyazev et al., 2016).

The attention of researchers is attracted to the study of serotonin transporter gene polymorphism, which is of great importance in the regulation of the serotoninergic system that affects mood, emotional regulation and behavior (Cools et al., 2008). Serotonin transporter (5-HTT) performs an important function – serotonin reuptake, thereby controlling the level of serotonin in the synaptic cleft and determining the intensity and duration of its action. The 5-HTT gene polymorphism has been extensively studied and represents a variation in the promoter region of the gene. This polymorphism has two major alleles: long (L) and short (S). The difference in promoter region length affects the transcription efficiency of the gene, i. e. the amount of transporter protein synthesized. The S allele is associated with reduced expression of the 5-HTT gene, resulting in less serotonin transporter in the synapse (Lesch et al., 1996). The long L allele contains a/g single nucleotide polymorphism. According to (Hu et al., 2006), the Lg allele is functionally similar to the S allele. The La/La genotype is classified as LL (high level of transcriptional efficiency), the La/S and La/Lg genotypes are classified as LS (intermediate level of transcriptional efficiency), and the Lg/S, Lg/Lg and S/S genotypes are classified as SS (low level of transcriptional efficiency) (Hu et al., 2006)

Studies have found an association between 5-HTT gene polymorphisms and susceptibility to various psychiatric disorders including MDD, post-traumatic stress disorder (PTSD), and suicidal behavior. It was found that individuals with at least one S allele showed increased sensitivity to stressful situations and negative emotions and were more likely to experience symptoms of MDD (Caspi et al., 2003) and PTSD (Koenen et al., 2009) in the presence of adverse factors. It should be noted that other studies have not confirmed associations between psychiatric disorders and the 5-HTT gene polymorphism (Risch et al., 2009). Also, in some studies, it was shown that carriers of the S allele are more prone to impulsive behavior (Walderhaug et al., 2010), have a higher expression of the personality trait neuroticism, the opposite pole of which is emotional stability (Greenberg et al., 2000), and also showed a reduced ability to cognitive control in the Stop Signal task (Landro et al., 2015).

fMRI study revealed that carriers of the S allele in response to negative stimuli showed increased activation of the amygdala, a brain structure responsible for detecting threats and experiencing emotions of fear and anxiety (Adolphs, 2008). At the same time, carriers of the S allele showed decreased activity in the ventromedial prefrontal cortex, a brain structure responsible for emotion regulation processes (Rao et al., 2007). In another study involving 23 women, the volume of the lateral prefrontal cortex, a brain structure involved in emotion regulation and cognitive control, was negatively associated with attention to emotional stimuli (both negative and positive) only in carriers of the S allele, but no polymorphism was associated with the volume of the amygdala and medial prefrontal cortex (Beevers et al., 2010). In a study of children and adolescents using the functional connectivity method, lower connectivity of the posterior node of the DMN with the superior medial prefrontal cortex was found in SS genotype carriers compared to L allele carriers (Wiggins et al., 2012). In a study by A. Meyer-Lindenberg (2009), it was found that indicators of functional connectivity in the brain are more reliable predictors of the effect of 5-HTT gene polymorphism than indicators of activity of individual brain regions (Meyer-Lindenberg, 2009). An fMRI study by J. Wiggins and colleagues (2012) examined the severity of connectivity between the anterior and posterior DMN nodes in children and adolescents and its association with age in groups dependent on serotonin transporter gene polymorphism (Wiggins et al., 2012).

In this study, we will not limit ourselves to examining connectivity between DMN nodes, but will analyze DMN connections within the cortex in the resting state depending on the serotonin transporter gene polymorphism in adult study participants. The aim of the study was to examine the connectivity features of the DMN measured from EEG data while at rest in relation to the serotonin transporter gene polymorphism.

## Materials and methods

Study participants. This study included 53 right-handed volunteers (32 females and 21 males) with normal or corrected-tonormal vision (mean age = 28 years, standard deviation = 10). Prior to the study, participants completed a questionnaire in which they answered questions about their mental and physical health, general well-being, and psychoactive substance use prior to the study. Women additionally answered questions about their current menstrual cycle phase and hormonal contraceptive use. Exclusion criteria were the presence of mental illness, head injuries, and use of narcotic, psychoactive, and hormonal substances. Participants were informed about the study methods and then signed an informed consent to participate in the experiment. The study was organized in accordance with the ethical standards established by the Declaration of Helsinki and was approved by the local bioethical committee of Scientific Research Institute of Neurosciences and Medicine (SRINM, Novosibirsk).

Genotyping. DNA was isolated from buccal epithelial cells using the Biosilica isolation kit (Russia). The La, Lg, and S alleles of the 5-HTT polymorphism were determined in DNA samples by polymerase chain reaction with primers: 5′-gagggactgagctggacaacccac-3′ and 5′-gcgttgccgctctgaattgc- 3′ (Lesch et al., 1996). The resulting products were separated by agarose gel electrophoresis. The sizes of the L and S alleles for the 5-HTT gene were 529 and 489 bp. To detect the La and Lg alleles, hydrolysis of the amplification products was carried out for three hours by MspI endonuclease. Thereafter, the product sizes were 340, 127, and 62 bp for the La allele and 174, 166, 127, and 62 bp for the Lg allele. The sample included 17 LL, 28 LS, and 8 SS carriers

EEG recording. For EEG recording, a Brain Products (Germany) multichannel biopotential amplifier was used, which was equipped with a cap containing 128 electrodes; one of the electrodes was used to record the oculogram. The electrodes were arranged according to the international 10–5 system. The bandwidth was between 0.1 and 100 Hz and the sampling rate was 1,000 Hz. Electrode Cz was used as a reference.

Data analysis. Artefacts were manually removed using independent component analysis (ICA) under visual control. Consistently with previous studies of oscillatory resting state networks (Knyazev et al., 2016; Bocharov et al., 2021, 2022), analysis was performed in the delta frequency range. After removing artefacts, EEG data were filtered in the delta frequency range (1–4 Hz) using the Butterworth filter and the filtfilt function. The boundary element model was used as the head model (Fuchs et al., 2001). The cortical grid used in the study contained 5,124 vertices and was created using a template based on the Montreal Neurological Institute (MNI) brain model. Localization of cortical sources of bioelectrical activity was performed using the beamformer method (Van Veen et al., 1997) with SPM-12 (https://www.fil.ion.ucl. ac.uk/spm/). Covariance matrices for the analysis were calculated on resting state EEG data recordings of 5 minutes. The orthogonalization method was used to correct for signal leakage that may arise due to insufficient spatial resolution of the source localization method (Brookes et al., 2011; Hipp et al., 2012).

After orthogonalization, a Hilbert transform was applied and the signal envelope was extracted, which is a curve of signal amplitudes over time. Connectivity maps were calculated between the time series of the selected regions of interest (ROIs) and the time series of the rest of the brain voxels. The selection of ROIs was based on data from previous studies. The medial prefrontal cortex (–1, 49, –2), posterior cingulate cortex (–5, –53, 41), and left (–45, –71, 35) and right (45, –71, 35) lateral parietal lobes were selected for the DMN (Gusnard, Raichle, 2001).

A Fisher transform was applied to the Pearson correlation coefficients between the temporal activity of ROIs and the rest of the brain. The connectivity maps were spatially smoothed (FWHM 8 mm) and converted to NIFTI format. Second-level statistical analysis was performed using T-contrast to detect differences between groups (carriers of the S allele vs carriers of the LL genotype). To assess the statistical significance of the detected effects, a double threshold was used: at the voxel level (p <0.005) and at the cluster level (cluster size greater than 100 voxels).

## Results

Statistical analysis (T-contrast) of DMN connectivity in two groups (S allele carriers vs LL genotype carriers) was performed in the SPM 12 program

The Figure A shows the results comparing DMN connectivity in S allele carriers and LL genotype carriers. So, it was found that S allele carriers had greater DMN connectivity in the left hemisphere with precuneus (BA (Brodmann Area) 19, x = –17, y = –82, z = 41, cluster size = 166, T = 3.10, p = 0.001) and with a cluster in the left hemisphere encompassing the posterior cingulate cortex (BA 30, x = –29, y = –70, z = 15, cluster size = 943, T = 3.08, p = 0.001), cuneus (BA 30, x = –25, y = –72, z = 7, cluster size = 943, T = 3.32, p < 0.001), and middle occipital gyrus (BA 19, x = –29, y = –70, z = 15, cluster size = 943, T = 3.05, p = 0.001), and with a cluster in the right hemisphere encompassing the posterior cingulate cortex (BA 30, x = 17, y = –54, z = 9, cluster size = 341 T = 3.19, p = 0.001) and the parahippocampal gyrus (BA 19, x = 17, y = –46, z = –3, cluster size = 341, T = 3.03, p = 0.001) (see the Figure A).

**Fig. 1.**
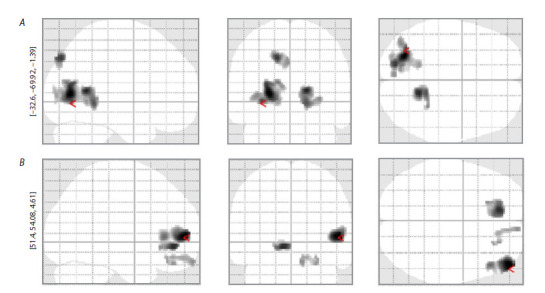
Results of statistical analysis (T-contrast) of the connectivity of the DMN between the group of carriers of the S allele and the group of carriers of the LL genotype. A – T-contrast DMN connectivity in S allele carriers is greater than DMN connectivity in LL genotype carriers; B – T-contrast DMN connectivity in LL genotype carriers is greater than DMN connectivity in S allele carriers.

The Figure B shows the results of the T-contrast of DMN connectivity in carriers of the LL genotype more than in carriers of the S allele. LL genotype carriers had more DMN connectivity with the medial frontal gyrus (BA 10, x = –11, y = 40, z = –7, cluster size = 251, T = 2.97, p = 0.002), with the superior frontal gyrus (BA 11, x = 9, y = 62, z = –21, cluster size = 110, T = 2.76, p = 0.003) and with a cluster located in the right dorsolateral prefrontal cortex (BA 10, x = 51, y = 54, z = 5, cluster size = 473, T = 3.06, p = 0.001) (see the Figure B).

According to (Raichle, 2015), anterior and posterior DMN nodes may have different functional significance. To understand the contribution of the anterior and posterior DMN nodes to the identified effects, we performed comparisons for the anterior and posterior DMN nodes separately in LL genotype carriers and S allele carriers.

Comparison of anterior DMN connectivity in S allele carriers, which was greater than in LL genotype carriers, showed no significant effects. Whereas LL genotype carriers had higher anterior node DMN connectivity with posterior cingulate cortex compared to S allele carriers (BA 23, x = 2, y = –44, z = 29, cluster size = 1,800, T = 3.77, p < 0.001), with the right superior frontal gyrus (BA 10, x = 23, y = 60, z = –3, cluster size = 581, T = 3.08, p = 0.001) and the medial frontal gyrus (BA 6, x = –11, y = –12, z = 57, cluster size = 292, T = 3.05, p = 0.001).

S allele carriers compared to LL genotype carriers had greater connectivity of the posterior DMN node with a cluster in the right hemisphere encompassing the posterior cingulate cortex (BA 30, x = 17, y = –56, z = 9, cluster size = 249, T = 2.95, p = 0.002) and the parahippocampal gyrus (BA 19, x = 19, y = –46, z = –5, cluster size = 249, T = 2.79, p = 0.002), and with a cluster in the left hemisphere encompassing the posterior cingulate cortex (BA 30, x = –29, y = –70, z = 15, cluster size = 782, T = 3.08, p = 0.001) and cuneus (BA 17, x = –11, y = –76, z = 13, cluster size = 782, T = 3.10, p = 0.002).

LL genotype carriers compared to S allele carriers had greater connectivity of the posterior DMN node with a cluster of voxels spanning the right dorsolateral prefrontal cortex (BA 46, x = 43, y = 30, z = 17, cluster size = 234, T = 2.77, p = 0.003).

## Discussion

In our study, the pattern of DMN connectivity with the precuneus, posterior cingulate cortex, parietal and occipital cortex was revealed in carriers of the S allele as compared to representatives of the LL genotype. The precuneus and posterior cingulate cortex are known to be part of the neural substrate of self-consciousness and form the posterior node of the DMN (Raichle et al., 2001; Utevsky et al., 2014). It has been repeatedly shown that the DMN is associated with internally directed thought processes and increases its activity during self-referential processing, autobiographical memories, thinking about the future, and making plans (Knyazev, 2013; Menon, D’Esposito, 2022). According to (Raichle, 2015), there is a functional separation between the anterior and posterior DMN nodes and the posterior DMN node is predominantly associated with processes of remembering past events (Raichle, 2015). It has also been shown in studies that joint activation of posterior cingulate cortex and precuneus has been observed during memory retrieval and reliving of traumatic events in patients with PTSD (Ramage et al., 2013; Thome et al., 2020). And under conditions of emotion regulation compared to passive observation of emotional stimuli, reduced activation of posterior cingulate cortex and precuneus was observed in patients with PTSD and in a group of healthy subjects (Nicholson et al., 2022)

Interestingly, in addition to increased DMN connectivity in the precuneus and posterior cingulate cortex, S allele carriers were also characterized by increased connectivity to the parietal and occipital cortex, which are part of the visual association cortex. In studies, the thickness of the visual associative cortex has been found to correlate with the severity of depression and anxiety (Peterson et al., 2009). It has also been shown that the visual associative cortex is involved in rumination processes (Cooney et al., 2010).

In carriers of the S allele compared to carriers of the LL genotype, an increase in DMN connectivity with the right parahippocampal gyrus, which is part of the temporal cortex, was found. The DMN is known to be associated with introspective thinking activity, while the temporal cortex and parahippocampal gyrus are involved in memory retrieval processes (Buckner, DiNicola, 2019). In a previous study, we found a positive association of depression symptom severity with the pattern of DMN connectivity with the right temporal cortex (Bocharov et al., 2021). Also, in a subsequent study, we found that an increase in this connectivity pattern correlated with the severity of intrusive thoughts: rumination and preference for a non-adaptive emotion regulation strategy – thought suppression (Bocharov et al., 2022). It should be noted that in the study (Rassin, 2003), it was found that when using such a strategy, the desire to suppress intrusive thoughts paradoxically leads to their strengthening (Rassin, 2003). It is known that carriers of the S allele are more prone to develop MDD and have difficulties in regulating their emotional state (Caspi et al., 2003; Walderhaug et al., 2010). It can be assumed that the identified pattern of higher DMN connectivity with the posterior node of the DMN, posterior associative cortex and right temporal cortex (parahippocampal gyrus), which is characteristic of S allele carriers, may be associated with and/ or predispose to the occurrence of intrusive thoughts and may underlie the predisposition to the emergence and maintenance of symptoms of depression.

In contrast to S allele carriers, who showed increased connectivity of the posterior node of the DMN, LL genotype carriers had higher DMN connectivity in the anterior node: with the medial frontal gyrus and with the superior frontal gyrus, which is part of the ventromedial frontal cortex. According to (Raichle, 2015), the structures of the anterior node of the DMN may have different functional importance, so the dorsomedial prefrontal cortex is involved in self-referential processes, while the ventromedial part of the frontal cortex is involved in the processes of social behavior, motivation, mood and emotional processing. A study (Rao et al., 2007) found reduced activation of the ventromedial frontal cortex and increased activation of the amygdala in response to negative stimuli in carriers of the S allele compared to carriers of the LL genotype. Dysfunction of the ventromedial frontal cortex is thought to play a key role in the pathogenesis of affective and anxiety disorders. It is assumed that insufficient activity of the ventromedial frontal cortex may lead to impaired inhibition of the activity of the amygdala and, as a consequence, to pathologically increased levels of negative affect (Motzkin et al., 2015).

Notably, LL genotype carriers were characterized by increased connectivity of the anterior DMN node with the posterior cingulate cortex, which is part of the posterior DMN node, i. e. connectivity between the anterior and posterior DMN nodes was increased. To some extent, this result is consistent with the study (Cha et al., 2018). In it, they found that carriers of genotypes with low transcriptional efficiency had reduced connectivity between the superior frontal gyrus, which is part of the anterior DMN node, and the posterior DMN node. Reduced connectivity between these structures mediated increased impulsivity in SS genotype carriers (Cha et al., 2018).

In addition, our study revealed that carriers of the LL genotype were characterized by increased connectivity of the posterior node of the DMN, involved in autobiographical memory processes, with the right dorsolateral prefrontal cortex, responsible for cognitive control and regulatory functions (Scult et al., 2017). Thus, it has been shown in studies that the right dorsolateral prefrontal cortex is often used as a target area for the application of transcranial magnetic stimulation (TMS) in the treatment of PTSD, obsessive-compulsive disorder, and addictions. In a study conducted by B.D. Greenberg and U. Ziemann (1998), it was shown that exposure with TMS to an area of the right dorsolateral prefrontal cortex in patients with obsessive-compulsive disorder promoted a reduction in compulsive symptoms and improved mood. H. Kober and colleagues (2010) found that in smokers, decreased cravings were associated with increased activity of the dorsolateral prefrontal cortex (Kober et al., 2010). A study conducted by H. Cohen and colleagues (2004) demonstrated the effectiveness of TMS in reducing PTSD symptoms and the importance of this brain region in the regulation of stress and emotional responses (Cohen et al., 2004). According to a study (Wu et al., 2020), stimulation of the right dorsolateral prefrontal cortex of the brain can improve a person’s self-regulatory abilities, especially in the context of managing one’s desires and urges, as well as in the regulation of negative emotions (Wu et al., 2020). It is conceivable that in carriers of the LL genotype, who are known to be more resistant to stress and the development of depression (Caspi et al., 2003; Koenen et al., 2009) and the manifestation of impulsive behavior (Walderhaug et al, 2010), increased DMN connectivity with brain structures involved in cognitive regulation processes (dorsolateral and ventromedial prefrontal cortex) may contribute to more effective regulation of DMN processes related to autobiographical memories.

## Conclusion

The revealed features of DMN connectivity in the resting state in carriers of LL genotypes and carriers of the S allele contribute to the understanding of the links between serotonin transporter gene polymorphism and predisposition to affective disorders. Carriers of the S allele compared to carriers of the LL genotype showed a pattern of higher connectivity of the posterior DMN, involved in autobiographical memory processes, with the precuneus, posterior cingulate and visual association cortex, and the right parahippocampal gyrus, which may predispose to the emergence and maintenance of obsessive thoughts. Whereas LL genotype carriers had higher DMN connectivity in the anterior node of the DMN: with the medial frontal gyrus, ventromedial frontal cortex, and posterior cingulate cortex, which is part of the posterior node of the DMN. Also, carriers of the LL genotype had higher resting-state connectivity of the posterior DMN with the cluster encompassing the right dorsolateral cortex compared to carriers of the S allele. It can be assumed that increased DMN connectivity with brain structures involved in cognitive regulation processes (dorsolateral and ventromedial prefrontal cortex) may contribute to the regulation of DMN processes associated with autobiographical memories

## Conflict of interest

The authors declare no conflict of interest.
